# Rapidly increasing prostheto-prosthetic pseudo aneurysm 26 years after cabrol procedure in a patient with Marfan syndrome: a case report

**DOI:** 10.11604/pamj.2023.45.100.36913

**Published:** 2023-06-22

**Authors:** Othmane Haddani, Besart Cuko, Frederic Vanden Eynden

**Affiliations:** 1 Department of Cardiac Surgery, Erasme University Hospital, *Université Libre de Bruxelles*, Brussels, Belgium,; 2 Department of Cardiology and Cardio-Vascular Surgery, *Hôpital Cardiologique de Haut-Lévèque*, Bordeaux University Hospital, Pessac, France

**Keywords:** Cabrol procedure, prostheto-prosthetic pseudoaneurysm, Marfan syndrome, reoperation, case report

## Abstract

A 63-year-old female with Marfan syndrome had undergone an initial operation of replacement of the ascending aorta and aortic valve with a composite graft and reconstruction of the coronary artery by the Cabrol procedure for aortic root dilatation and aortic valve regurgitation. During a follow-up of 16 years, a decreased ejection fraction was observed on transthoracic echocardiography with the onset of chest pain and dyspnea. Computer tomography angiography revealed a prostheto-prosthetic pseudoaneurysm, initially measured 21x16x23 mm, rapidly increased at 1-year follow-up at 27x24x33 mm. Coronary angiography showed the presence of turbulent flow inside the pseudoaneurysm with a decreased coronary perfusion. We resected the pseudoaneurysm and a new prostheto-prosthetic anastomosis was performed. The postoperative course was uneventful without any complications. We report this case because in literature there has been few reports regarding prostheto-prosthetic pseudoaneurysm after Cabrol procedure.

## Introduction

Cabrol procedure is an alternative of classical surgical methods of Bentall DeBono procedure for the treatment of annulo-aortal ectasia. The surgical ascending aorta repair according to the Cabrol procedure involves a composite aortic graft and a prosthetic conduit that connects the coronary ostia are anastomosed to the aortic graft. Pseudoaneurysm after Cabrol procedure is a rare condition. We report an unusual case of a 63-year-old female with Marfan syndrome operated by the Cabrol procedure with diagnosis of rapidly increasing prostheto-prosthetic pseudoaneurysm.

## Patient and observation

**Patient information:** a 63-year-old female with Marfan syndrome was admitted to our institution for dyspnea and chest pain on sustained effort. Her medical history included previous cardiac surgery by the Cabrol procedure for aortic root dilatation and aortic valve regurgitation with a strict follow-up for a known prostheto-prosthetic pseudoaneurysm.

**Clinical findings:** on physical examination the patient had normal blood pressure and normal peripheral oxygen saturation. Cardio-pulmonary auscultation and laboratory examination was also normal.

**Figure 1:**
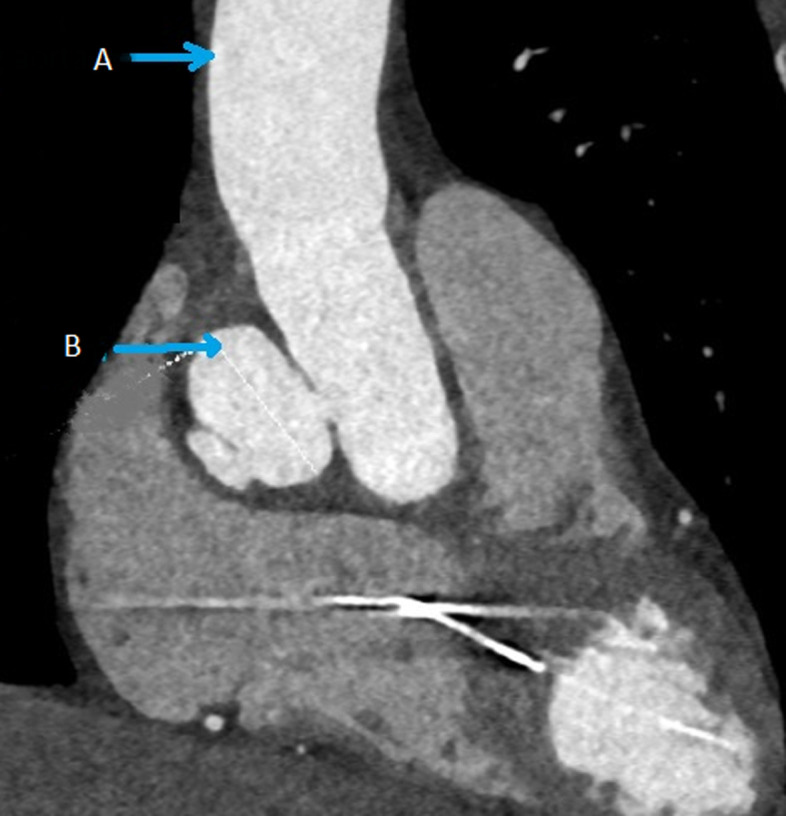
pre-operative computed tomography angiography scan showing prostheto-prosthetic pseudoaneurysm: A) ascending aorta; B) 3.3 cm pseudo aneurysm

**Figure 2:**
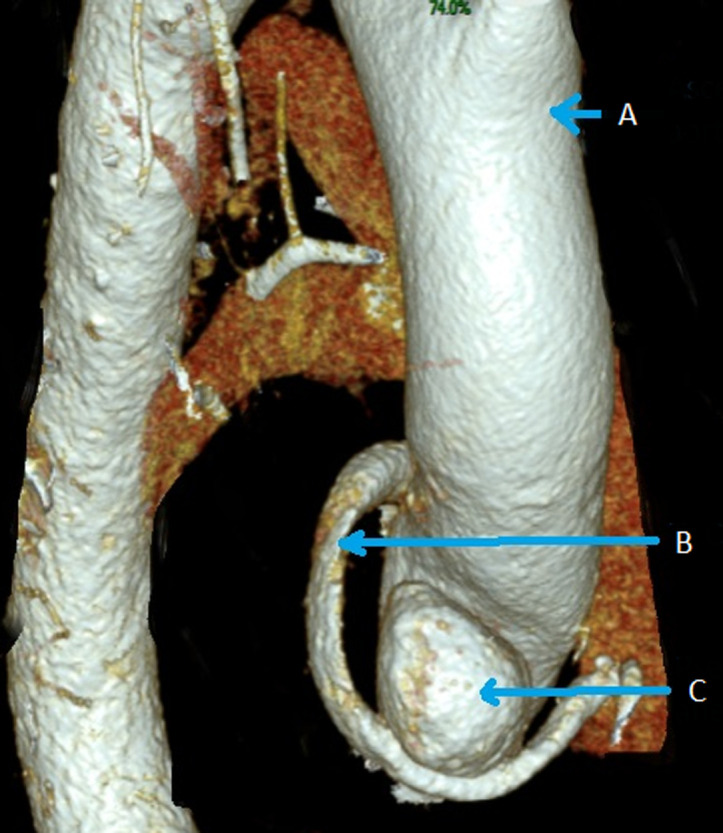
pre-operative computed tomography angiography scan showing rapidly increasing prostheto-prosthetic pseudoaneurysm: A) ascending aorta; B) T-Cabrol tube; C) pseudo aneurysm

**Diagnostic assessment:** the 12-lead electrocardiogram showed normal sinus rhythm. Transthoracic echocardiography (TTE) showed a decreased ejection fraction compared to the last TTE, respectively from 55-60% to 45-50%. Computed tomography angiography (CTA) effectuated during the hospitalization demonstrated a rapidly increasing prostheto-prosthetic pseudoaneurysm compared to the CTA 1-year before admission, respectively 21x16x23 mm to 27x24x33 mm (Figure 1, Figure 2). Coronary angiography showed the presence of turbulent flow inside the pseudoaneurysm with a decreased coronary perfusion.

**Diagnosis:** rapidly increasing prostheto-prosthetic pseudoaneurysm in a symptomatic patient.

**Therapeutic interventions:** after heart team assessment we decided to reoperate the patient considering the high risk of rupture due to rapidly increasing of pseudoaneurysm dimension. Under general anesthesia a full sternotomy was performed. The cardiopulmonary bypass (CPB) was established by the aortic cannulation for the arterial line and right atrial cannulation for the venous line. After infusion of cardioplegia solution. Resection of the pseudoaneurysm and a new prostheto-prosthetic anastomosis was performed. Postprocedural recovery was uneventful with a good hemodynamic response.

**Figure 3:**
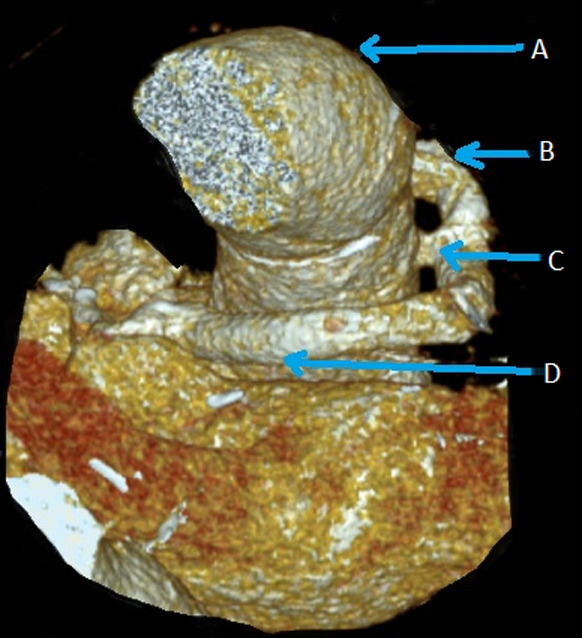
post-operative computed tomography angiography scan showing excellent surgical resultants: A) ascending aorta; B) right coronary tube; C) T-suture after redo; D) left coronary tube

**Follow-up and outcome of intervention:** the patient recovered uneventfully with no complications and was discharged at home after 12 days. Transthoracic echocardiography and CTA before discharge showed excellent surgical resultants (Figure 3).

**Informed consent:** patient's informed consent for the procedure and for data collection was obtained.

## Discussion

Proximal ascending aortic disease is a major cardiovascular manifestation in Marfan syndrome, frequently complicated by an acute aortic dissection [1]. In 1981, Cabrol *et al*. described the Cabrol procedure [2] as alternative of classical surgical methods of Bentall DeBono procedure [3] for the treatment of annulo-aortal ectasia. Since then long-term follow-up suggests that certain number of patients for various reasons requires reoperation through sternotomy.

In the literature, Mesana *et al*. [4] reported that 15% of ascending aorta prosthetic replacements were associated with false aneurysms. The most frequent causes are technical errors, infections, and dissection of the native aorta [5]. Kitamura *et al*. [6] reported that the most common complication after a Cabrol procedure was a coronary complication. Sugawara *et al*. reported a case report of pseudoaneurysm in the ascending aorta and the residual aortic dissection after 5 years of Cabrol procedure in a Marfan patient [7]. Tanaka *et al*. described a case of graft-to-graft anastomosis pseudoaneurysm on the modified Cabrol coronary reattachment technique after aortic root replacement [8].

To our knowledge, this is the first care report of a pseudoaneurysm 26 years after Cabrol procedure in a Marfan patient. We decided to reoperate due to the rapidly increasing pseudoaneurysm dimension in a symptomatic patient. Postprocedural recovery was uneventful, and the patient was discharged at home totally asymptomatic.

## Conclusion

Aortic root surgery can be complicated by a pseudoaneurysm between the prosthetic tissue and the patient's native tissue, especially in patients with Marfan syndrome. In our case, we observed an unusual pseudoaneurysm between two prosthetic tissues which is a rare and led to a reoperation of the patient. Regular follow-up is important in aortic root surgery, especially in Marfan syndrome patients, even after long-term surgery.

## References

[1] Kazui T, Yamashita K, Terada H, Washiyama N, Suzuki T, Ohkura K (2003). Late reoperation for proximal aortic and arch complications after previous composite graft replacement in Marfan patients. Ann Thorac Surg.

[2] Cabrol C, Pavie A, Gandjbakhch I, Villemot JP, Guiraudon G, Laughlin L (1981). Complete replacement of the ascending aorta with reimplantation of the coronary arteries: new surgical approach. J Thorac Cardiovasc Surg.

[3] Bentall H, De Bono A (1968). A technique for complete replacement of the ascending aorta. Thorax.

[4] Mesana TG, Caus T, Gaubert J, Collart F, Ayari R, Bartoli J (2000). Late complications after prosthetic replacement of the ascending aorta: what did we learn from routine magnetic resonance imaging follow-up?. Eur J Cardiothorac Surg.

[5] Greco E, Santamaria V, D'Abramo M, Totaro M, Frati G, Miraldi F (2020). Get out of trouble during redo surgery for false aneurysm of the ascending aorta. Asian Cardiovasc Thorac Ann.

[6] Kitamura T, Kigawa I, Fukuda S, Miyairi T, Takamoto S (2011). Long term results with the Cabrol aortic root replacement. Int Heart J.

[7] Sugawara Y, Shimakura T, Kihara S, Tanaka S, Saitoh N, Imamaki M (1998). [A combination of reoperation for pseudoaneurysm following the Cabrol procedure and total aortic arch replacement in a patient with Marfan syndrome--a case with an aberrant right subclavian artery]. Jpn J Thorac Cardiovasc Surg.

[8] Tanaka A, Al-Rstum Z, Zhou N, Hassan M, Sandhu HK, Miller CC (2020). Feasibility and Durability of the Modified Cabrol Coronary Artery Reattachment Technique. Ann Thorac Surg.

